# Future distinguishing competencies of baccalaureate-educated registered nurses in nursing homes

**DOI:** 10.1186/1472-6955-14-S1-S10

**Published:** 2015-10-08

**Authors:** Ramona Backhaus, Hilde Verbeek, Erik van Rossum, Elizabeth Capezuti, Jan PH Hamers

**Affiliations:** 1 Department of Health Services Research, Maastricht University, Maastricht, The Netherlands

## Background

In the future, working in nursing homes is likely to become more complex due to a tendency towards more personal choice, an increased complexity of residents, and technological innovations. An increase in bachelor-educated registered nurses (BRNs) might lead to an improvement in quality of life and quality of care for nursing home residents, as BRNs may be better able to deal with the increased complexity, and could have a supervisory role for less educated staff.

## Aims

As BRNs are an especially scarce resource in nursing homes, obtaining more information on how to allocate them in the nursing home is desirable. To allocate them in an optimal way, the aim is to reach consensus on their future distinguishing competencies first.

## Methods

Thirty-one international experts, identified through literature and our professional network, participated in a two-round web-based survey and an additional in-person focus group.

## Results

Experts reached consensus on 16 desirable future distinguishing competencies. Our findings suggest the importance of competencies beyond those related to traditional nurse expert roles, e.g. being a team leader, role model and coach within the nursing team. BRNs are expected to serve as advocates of resources in the facility and to be able to bring the right team to the client, maintaining accessibility to care.

## Conclusions

BRNs should be allocated in a way that allows them to act in a liaison role among nursing home residents, family members, and the multidisciplinary nursing home staff. In the future, particular attention should be paid to the development of leadership and coaching competencies, as these competencies are not explicitly covered in most current training programmes.

